# The Role of EGFR/PI3K/Akt/cyclinD1 Signaling Pathway in Acquired Middle Ear Cholesteatoma

**DOI:** 10.1155/2013/651207

**Published:** 2013-11-07

**Authors:** Wei Liu, Hongmiao Ren, Jihao Ren, Tuanfang Yin, Bing Hu, Shumin Xie, Yinghuan Dai, Weijing Wu, Zian Xiao, Xinming Yang, Dinghua Xie

**Affiliations:** ^1^Department of Otolaryngology, Head and Neck Surgery, The Second Xiangya Hospital, Central South University, 139 Middle Renmin Road, Changsha, Hunan 410011, China; ^2^Department of Pathology, The Second Xiangya Hospital, Central South University, Changsha, Hunan 410011, China

## Abstract

Cholesteatoma is a benign keratinizing and hyper proliferative squamous epithelial lesion of the temporal bone. Epidermal growth factor (EGF) is one of the most important cytokines which has been shown to play a critical role in cholesteatoma. In this investigation, we studied the effects of EGF on the proliferation of keratinocytes and EGF-mediated signaling pathways underlying the pathogenesis of cholesteatoma. We examined the expressions of phosphorylated EGF receptor (p-EGFR), phosphorylated Akt (p-Akt), cyclinD1, and proliferating cell nuclear antigen (PCNA) in 40 cholesteatoma samples and 20 samples of normal external auditory canal (EAC) epithelium by immunohistochemical method. Furthermore, *in vitro* studies were performed to investigate EGF-induced downstream signaling pathways in primary external auditory canal keratinocytes (EACKs). The expressions of p-EGFR, p-Akt, cyclinD1, and PCNA in cholesteatoma epithelium were significantly increased when compared with those of control subjects. We also demonstrated that EGF led to the activation of the EGFR/PI3K/Akt/cyclinD1 signaling pathway, which played a critical role in EGF-induced cell proliferation and cell cycle progression of EACKs. Both EGFR inhibitor AG1478 and PI3K inhibitor wortmannin inhibited the EGF-induced EGFR/PI3K/Akt/cyclinD1 signaling pathway concomitantly with inhibition of cell proliferation and cell cycle progression of EACKs. Taken together, our data suggest that the EGFR/PI3K/Akt/cyclinD1 signaling pathway is active in cholesteatoma and may play a crucial role in cholesteatoma epithelial hyper-proliferation. This study will facilitate the development of potential therapeutic targets for intratympanic drug therapy for cholesteatoma.

## 1. Introduction

Cholesteatoma is a benign keratinizing and hyperproliferative squamous epithelial lesion of the temporal bone which gradually expands and causes serious complications by destruction of nearby bony structures. Erosion of the ossicular chain and bony labyrinth may cause hearing loss, facial paralysis, labyrinthine fistulae, and brain abscess. In general, squamous epithelial cells of cholesteatoma are characterized by uncoordinated proliferation, migration, aggressiveness, and recurrence [1–4]. However, insight into its exact underlying cellular and molecular mechanisms remains incomplete. Clinically, acquired cholesteatoma is usually related to chronic suppurative inflammation of the middle ear, which plays an important role in the etiopathogenesis of cholesteatoma [5]. Recently, many authors have demonstrated that cytokines and mediators which are released in the inflammatory responses may promote the formation and progression of cholesteatoma by stimulating migration and proliferation of keratinocytes in the tympanic membrane and deep part of external auditory canal (EAC) [5–10].

Epidermal growth factor (EGF) is one of the most important cytokines which has been shown to play a critical role in cholesteatoma [11, 12]. Increased EGF expression has been demonstrated in cholesteatoma epithelium [13, 14], and high EGF levels have also been shown in cholesteatoma debris and subepithelial tissue [15]. However, the exact mechanisms of EGF in the pathogenesis of cholesteatoma remain unclear. Recently, some downstream targets of EGF have been identified, and among them phosphatidylinositol 3-kinase (PI3K)/Akt signaling pathway is one of the most important kinase cascades that mediates a wide range of cellular functions such as survival, proliferation, migration, and differentiation [16]. The EGF receptor (EGFR) is a 170-kDa transmembrane glycoprotein that consists of intracellular, extracellular, and transmembrane domains [17]. The binding of EGF to the extracellular domain of EGFR promotes the activation of EGFR through autophosphorylation of their intracellular domains, which subsequently triggers the PI3K/Akt signaling pathway [18]. Activation of the PI3K/Akt signaling pathway can lead to cell proliferation and growth by regulating cyclinD1 expression [19]. Recent studies have shown that EGFR immunoreactivity was significantly increased in cholesteatoma epithelium when compared with normal EAC epithelium [20–23]. These results lead us to hypothesize that EGF in the inflammatory microenvironment of cholesteatoma may bind with EGFR and activate the EGFR/PI3K/Akt/cyclinD1 signaling pathway, which, in turn, promotes the abnormal proliferation of keratinocytes in cholesteatoma epithelium.

To test our hypotheses, firstly we examined the expressions of phosphorylated EGFR (p-EGFR), phosphorylated Akt (p-Akt), cyclinD1, and proliferating cell nuclear antigen (PCNA) in middle ear cholesteatoma and normal EAC skin specimens. Secondly, we developed a human external auditory canal keratinocytes (EACKs) cell culture model and investigated EGF-induced downstream signaling in EACKs.

## 2. Materials and Methods

### 2.1. Patients and Specimens

Forty samples of acquired cholesteatoma tissues diagnosed clinically and confirmed pathologically after middle ear surgery were obtained from the Department of Otolaryngology, Head and Neck Surgery, The Second Xiangya Hospital, Central South University, from January 2008 to October 2010. There were 23 males and 17 females with a mean age of 38.5 years (range 14–56 years). Twenty normal EAC skin samples obtained from patients undergoing myringoplasty for dry perforation and exploratory tympanotomy for diagnosis of middle ear disease served as controls. Specimens for hematoxylin-eosin (HE) staining and immunohistochemistry were immediately fixed in 10% buffered formalin and embedded in paraffin. All samples were analyzed independently by two experienced pathologists. The study was approved by the Ethics Committee of Central South University and informed consent was obtained from all of the patients.

### 2.2. Immunohistochemistry

Paraffin-embedded tissues were cut into 4 *μ*m thick sections. The sections were then deparaffinized in xylene and rehydrated in a graded ethanol series (100%, 95%, and 75%) before incubation for 10 min with 3% hydrogen peroxide solution. Antigen retrieval was performed in 10 mmol/L citrate buffer (PH 6.0) in a microwave oven for 15 min at 100°C. The sections were washed in phosphate-buffered saline (PBS) and preincubated with nonimmune goat blood serum for 15 min at room temperature. Then the sections were incubated with primary rabbit antibodies against p-EGFR (Tyr 1173; 1 : 100 dilution; Santa Cruz Biotechnology, USA), p-Akt (Ser 473; 1 : 50 dilution; Cell signaling Technology, USA), cyclinD1 (1 : 25 dilution; Cell signaling Technology, USA), and PCNA (1 : 100 dilution; Bioworld Technology, USA) at 4°C overnight in a humidified chamber. After incubation, each section was washed with PBS and incubated with the secondary biotinylated goat-anti-rabbit antibody (Zhongshan Goldenbridge Biotechnology, Beijing, China) for 15 min at room temperature. After being washed in PBS, the sections were incubated with streptavidin conjugated with horse radish peroxidase (Zhongshan Goldenbridge Biotechnology, Beijing, China). Freshly prepared 3,3′-diaminobenzidine (DAB, Sigma) was used as a substrate for peroxidase. Finally, the sections were counterstained with hematoxylin, dehydrated, and mounted. For the negative controls, PBS was used to substitute primary antibody.

### 2.3. Evaluation of Immunostaining

Both the percentage of stained cells and the intensity of staining were considered [24]. The percentage of positive cells was graded and scored as 0 for negative staining, 1 for <10%, 2 for 10%–50%, and 3 for >50% positive cells. Staining intensity was graded and scored as 0 for no staining, 1 for weak staining, 2 for moderate staining, and 3 for strong staining. The overall score for each specimen was obtained by multiplying the percentage score and the staining intensity score. The final results were recorded as negative (−) if the overall score was ≤2 and positive (+) if the overall score was ≥3.

### 2.4. Primary Cultures of EACKs

EACKs were obtained from external auditory canal skin (EACS) samples during exploratory tympanotomy surgery as described previously by Schmidt et al. [12] and Sanjuan et al. [25]. Briefly, tissue samples were rinsed with cold sterile PBS containing 100 IU/mL of penicillin and 100 ug/mL of streptomycin prior to removal of connective tissue. Then, the samples were cut into small pieces and incubated in 0.25% trypsin solution overnight at 4°C. Subsequently, epithelial sheets were dissected and dissociated with trypsin. Dissociation was stopped with trypsin inhibitor, and cells were then centrifuged for 5 minutes at 1000 rpm. After centrifugation, cells were cultured in defined keratinocyte-SFM (serum-free medium for keratinocytes; Invitrogen, USA) containing supplements (bovine pituitary extract, human epidermal growth factor; Invitrogen) in humidified CO_2_ at 37°C. All experiments utilized subconfluent, low-passage EACKs. The study was approved by the Ethics Committee of Central South University and informed consent was obtained from all of the patients.

### 2.5. Identification of EACKs by Immunofluorescence

The cells were cultured onto coverslips and then fixed with 4% paraformaldehyde solution followed by incubation with 0.2% Triton X-100 for 10 min to allow permeabilization. After incubation, the cells were washed three times in PBS and incubated in blocking buffer (3% bovine serum albumin). The cells were then incubated with primary mouse antibody against cytokeratins (1 : 150 dilution; Boster, China) at 37°C for 90 min. After incubation, the cells were washed in PBS and incubated with TRITC-labeled goat-anti-mouse secondary antibody (1 : 50 dilution; Boster, China) at 37°C for 60 min. Coverslips were finally mounted for observation. PBS was used to substitute the primary antibody (cytokeratins) as a negative control.

### 2.6. Cell Treatment

EACKs from passages two or three were used for the study. Defined keratinocyte-SFM with supplements was changed to medium without supplements for 24 h before treatment with EGF. In brief, EACKs were treated with 15 ng/mL of human recombinant EGF (Sigma, USA) for the indicated periods of time. For the inhibition experiments, EACKs were cultured in defined keratinocyte-SFM containing 250 nM of the EGFR inhibitor AG1478 (Cell signaling Technology, USA) for 30 min or 200 nM of the PI3K inhibitor wortmannin (Sigma, USA) for 1 h before treatment with EGF.

### 2.7. MTT Assay

The capacity for cellular proliferation was evaluated by MTT assay. Briefly, cells were seeded into 96-well plates and incubated in humidified CO_2_ at 37°C. 24 h later, the cells were stimulated with EGF (15 ng/mL) in the presence or absence of EGFR inhibitor (AG1478, 250 nmol/L) and PI3K inhibitor (wortmannin, 200 nmol/L) for the indicated periods of time. The cells were then incubated with 20 *μ*L of MTT solution for 4 h at 37°C. After removal of the culture medium, 150 *μ*L of dimethyl sulfoxide (DMSO) was added to solubilize the crystals. The cell growth curve was drawn according to the values of 490 nm wavelength light absorption (OD_490_). Cell survival rate was (%) = OD_treat_/OD_control_ × 100%.

### 2.8. Cell Cycle Analysis

EACKs were cultured in 6-well tissue culture plates and grown to 70% to 80% confluence. At predetermined time points after treatment, EACKs were harvested and fixed with ice-cold ethanol (70%). After fixation, the cells were centrifuged and washed with ice-cold PBS. Then, the cells were incubated with PBS containing propidium iodide (PI, 50 *μ*g/mL; Sigma, USA), RNase A (100 *μ*g/mL; Beyotime, China), and 0.1% Triton X-100 (Beyotime, China) and kept in the dark at 4°C for 30 min. The DNA content was determined by flow cytometry.

### 2.9. Western Blotting Analysis

After treatment, cell lysates were prepared and separated by SDS-PAGE gel and transferred to polyvinylidene difluoride membrane. Membranes were blocked with Tris-buffered saline (TBS, PH 7.4), containing 5% nonfat dry milk, and then incubated with primary antibodies overnight at 4°C. After being washed three times in TBS, the membranes were incubated with horseradish peroxidase-conjugated goat-anti-rabbit secondary antibody (1 : 1000; Santa Cruz Biotechnology, USA) for 1 h at room temperature. Proteins were detected by the enhanced chemiluminescence (ECL) method (Thermo, USA). The details about the primary antibodies were as follows: p-EGFR (Tyr 1173; 1 : 500 dilution; Santa Cruz Biotechnology, USA), p-Akt (Ser 473; 1 : 2000 dilution; Cell signaling Technology, USA), cyclinD1 (1 : 500 dilution; Bioworld Technology, USA), EGFR (1 : 500 dilution; Bioworld Technology, USA), and Akt (1 : 500 dilution; Bioworld Technology, USA).

### 2.10. Statistical Analysis

Protein expression patterns between cholesteatoma epithelium and normal EAC epithelium were analyzed with the Chi-square test (*χ* ^2^ test). Spearman's rank correlation test was used to calculate correlations. We also analyzed the differences between the treated cell groups and control cell groups using Student's *t*-test. Statistical analysis was performed by SPSS 16.0 statistical program (SPSS Inc., Chicago, IL, USA). A value of *P* < 0.05 was considered statistically significant.

## 3. Results

### 3.1. Histopathological Findings

**Figure 1:**
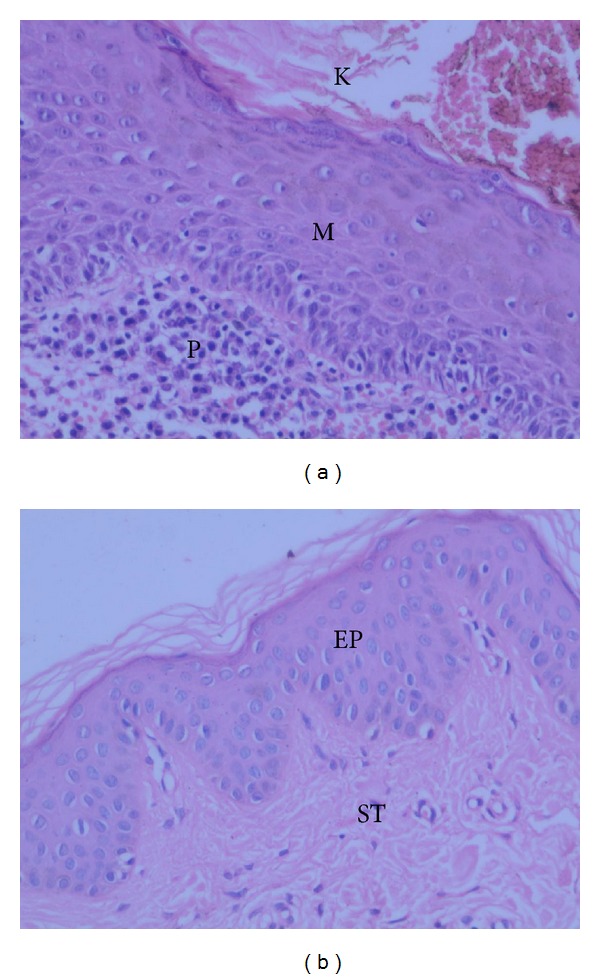
HE staining of human cholesteatoma (a) and normal EAC skin (b). In the figure, the keratin debris (K), matrix epithelium (M), and perimatrix subepithelial tissue (P) of cholesteatoma and the epithelium (EP) and subepithelial tissue (ST) of normal EAC skin are indicated. Original magnification: ×200.

HE staining showed that all cholesteatoma specimens consisted of three parts: matrix, perimatrix, and keratin debris (Figure 1(a)). In cholesteatoma specimens, inflammatory cells and newly formed blood vessels were always seen in the perimatrix. No signs of inflammation were observed in the normal EAC skin sections (Figure 1(b)).

### 3.2. Immunohistochemical Detection of p-EGFR, p-Akt, and cyclinD1 in Cholesteatoma Epithelium and Normal EAC Skin Epithelium

**Figure 2:**
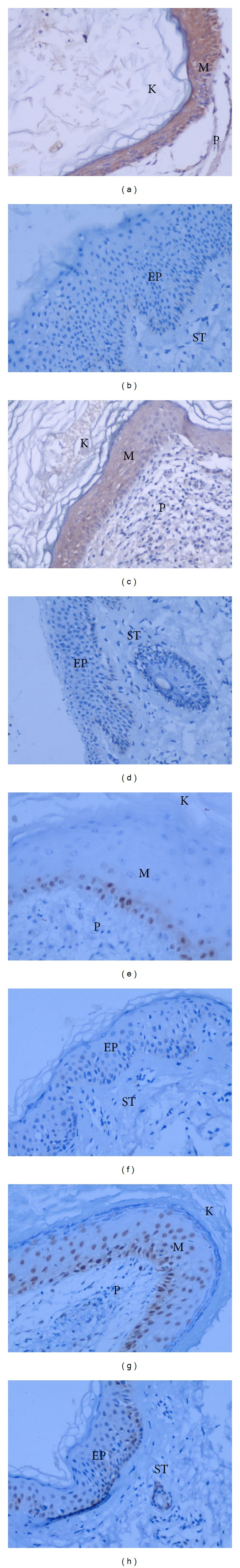
Immunohistochemical staining of p-EGFR ((a), (b)), p-Akt ((c), (d)), cyclinD1 ((e), (f)), and PCNA ((g), (h)) in paraffin sections of human cholesteatoma epithelium ((a), (c), (e), (g)) and normal EAC skin epithelium ((b), (d), (f), (h)). In the figure, the keratin debris (K), matrix epithelium (M), and perimatrix subepithelial tissue (P) of cholesteatoma and the epithelium (EP) and subepithelial tissue (ST) of normal EAC skin are indicated. Original magnification: ×200.

As shown in Figure 2, positive p-EGFR immunostaining was mainly observed in the cell cytoplasm and membrane of cholesteatoma epithelium in the basal and suprabasal layers (Figure 2(a)); p-Akt positive reactions were mainly localized in the cytoplasm of epithelium in the basal and suprabasal layers (Figure 2(c)); cyclinD1 expression was mainly observed in the nuclei and was localized in the basal layer of the epithelium (Figure 2(e)); PCNA positive reactions were mainly observed in the nuclei and was localized in the basal and suprabasal layers (Figure 2(g)). The positive rate of p-EGFR, p-Akt, cyclinD1, and PCNA expression was significantly increased in cholesteatoma epithelium (65.0%, 72.5%, 62.5%, and 80.0%, resp.) when compared with normal EAC epithelium (20.0%, 35.0%, 10.0%, and 45.0%, resp.) (Table 1; *P* < 0.01). In cholesteatoma epithelium, a significant positive correlation was observed between p-EGFR and PCNA expression and between the expressions of p-Akt and PCNA, cyclinD1, and PCNA, respectively (*P* < 0.01).

### 3.3. Establishment and Identification of Primary Cultures of EACKs

**Figure 3:**
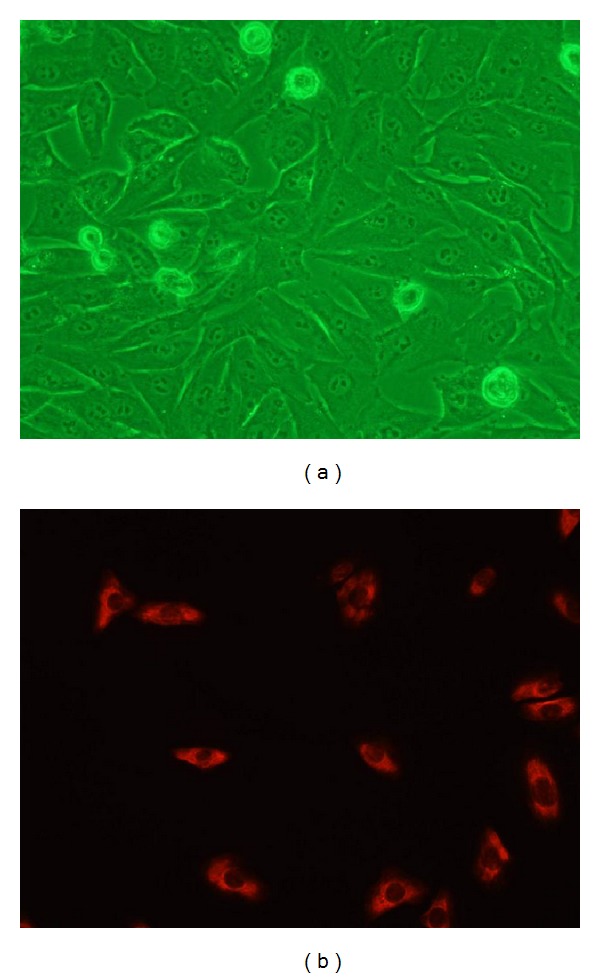
The cultured cells grew into monolayer and showed a polygonal “cobblestone-like” appearance (a). The cultured cells showed a positive reaction to cytokeratins by immunofluorescence, and the signal was detected in cytoplasm (b).

After 5–7 days of culture, we observed cell colonies made up of a few cells, and the cells gradually grew outwards from the colonies. After 20–22 days of culture, the cultured cells grew into monolayer and appeared polygonal with “cobblestone-like” appearance (Figure 3(a)). The cells were incubated with anti-cytokeratins antibody and showed a positive reaction to cytokeratins (Figure 3(b)). No signal was detected for the negative controls. The morphological and biological characteristics of primary cultures of EACKs in the present study were similar to those of typical keratinocytes.

### 3.4. Inhibition of EGFR/PI3K/Akt Pathway Downregulates EGF-Induced Proliferation of EACKs

**Figure 4:**
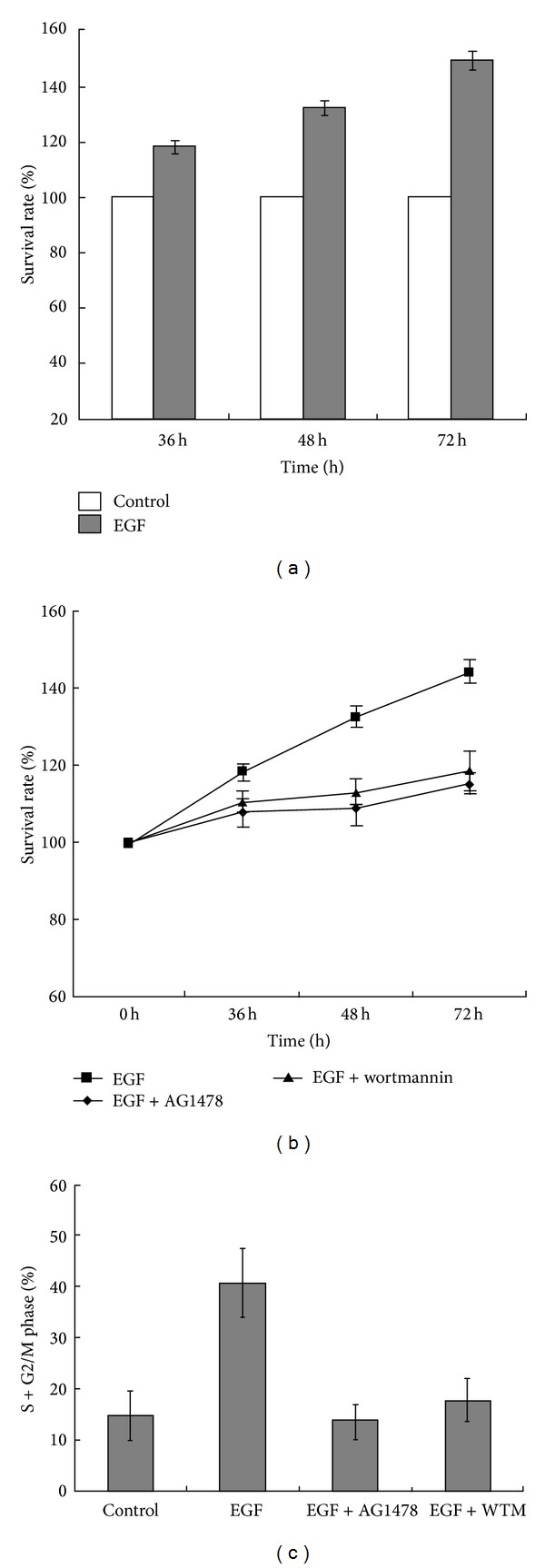
EACKs were treated with 15 ng/mL of EGF for the indicated periods of time. Cells proliferation was detected by MTT assay (a). EACKs were pretreated with AG1478 or wortmannin (WTM) for 1 h and then exposed to 15 ng/mL of EGF for the indicated periods of time. Cells proliferation was detected by MTT assay (b). EACKs were incubated with or without AG1478 or wortmannin (WTM) for 1 h and then exposed to 15 ng/mL of EGF for 72 h. Cells were harvested for cell cycle analysis by flow cytometry (c). Results were summarized as mean ± SD and represent at least three independent experiments.

As shown in the Figure 4(a), EGF (15 ng/mL) treatment significantly increased the proliferation of EACKs at all time points (Table 2; *P* < 0.05). We hypothesized that the EGFR/PI3K/Akt survival signaling pathway may be involved in EGF-induced proliferation of EACKs. Here, we investigate this by pretreatment of EACKs with EGFR inhibitor (AG1478, 250 nmol/L) and PI3K inhibitor (wortmannin, 200 nmol/L) for the indicated periods of time. According to the results of the MTT assay, we observed that the pretreatment of EACKs with AG1478 or wortmannin did significantly inhibit the cell proliferation of EACKs induced by EGF stimulation (Figure 4(b) and Table 2; *P* < 0.05). Our studies suggested that EGF might induce the cell proliferation of EACKs through the EGFR/PI3K/Akt signaling pathway.

### 3.5. Inhibition of EGFR/PI3K/Akt Pathway Downregulates EGF-Induced Cell Cycle Progression of EACKs from G0/G1 Phase to S Phase

In order to further explore the mechanism of EGF-induced cell proliferation of EACKs, flow cytometry was used to monitor cell cycle changes. As shown in Figure 4(c), EGF treatment markedly increased the percentage of EACKs cells in S+G2/M phase when compared with the control group (40.81 ± 6.9 versus 14.76 ± 4.8) (*P* < 0.05). However, the pretreatment of cells with AG1478 significantly reduced the percentage of cells in S+G2/M phase when compared with the EGF treatment group (13.61 ± 3.4 versus 40.81 ± 6.9) (*P* < 0.05), and the pretreatment of cells with wortmannin significantly reduced the percentage of cells in S+G2/M phase when compared with the EGF treatment group (17.72 ± 4.2 versus 40.81 ± 6.9) (*P* < 0.05). These results demonstrated that EGF might induce cell cycle progression of EACKs through the EGFR/PI3K/Akt signaling pathway.

### 3.6. EGF Promotes Phosphorylation of EGFR and Akt without Affecting the Total Levels of EGFR and Akt in EACKs

**Figure 5:**
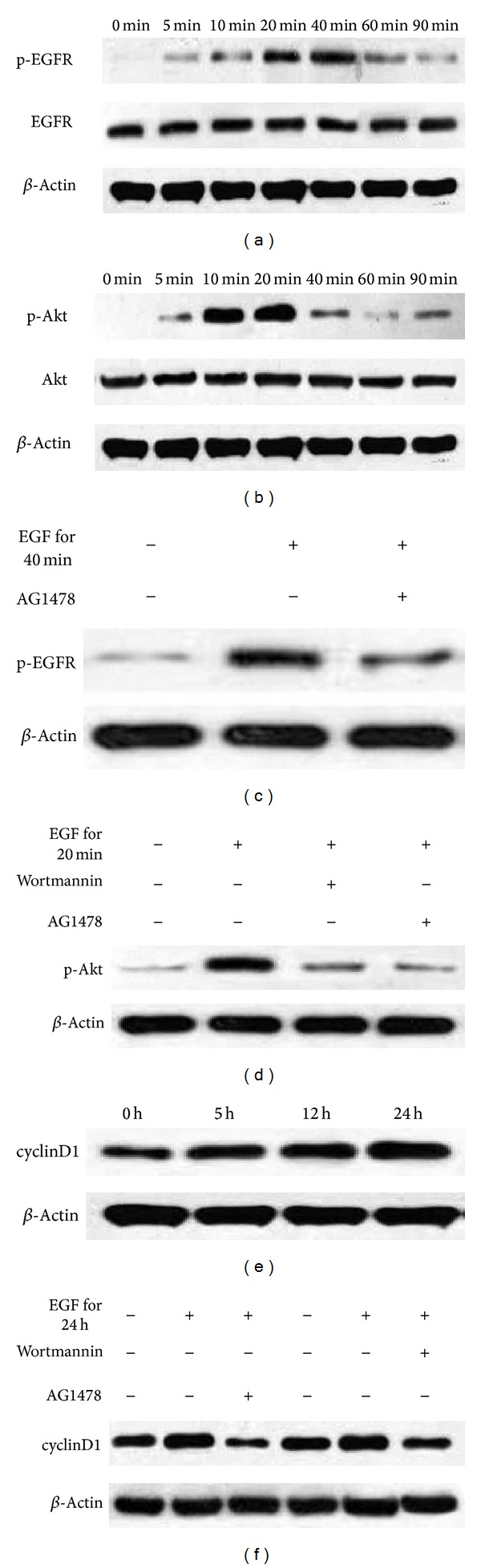
EGFR/PI3K/Akt signaling pathway is required for cyclinD1 upregulation in EGF-stimulated EACKs. Cells were treated with 15 ng/mL of EGF for the indicated periods of time ((a), (b), (e)) or incubated with or without AG1478 or wortmannin for 1 h and then exposed to 15 ng/mL of EGF for the indicated periods of time ((c), (d), (f)). After treatment, cells were harvested for Western blotting analysis using specific antibodies as indicated, and *β*-actin was used as an equal loading control.

In order to study the signaling pathways in EGF-induced cell cycle progression of EACKs, we determined activation/phosphorylation of EGFR and Akt by Western blotting analysis. As shown in Figure 5, EGF induced rapid phosphorylation of EGFR and Akt within 60 min, with peak phosphorylation of EGFR and Akt seen at 30 min and 20 min (Figures 5(a) and 5(b)), respectively. However, the total level of EGFR and Akt remained unchanged. To investigate whether EGFR activation is essential for the EGF-induced Akt phosphorylation, cells were pretreated with AG1478 for 30 min and then exposed to EGF. Here, we used the well-established PI3K inhibitor wortmannin as a positive control for Akt phosphorylation inhibition. The results demonstrated that AG1478 potently blocked EGF-induced phosphorylation of EGFR and Akt (Figures 5(c) and 5(d)). These findings indicate that EGFR activation is essential for the activation of PI3K/Akt signaling pathway in EGF-stimulated EACKs.

### 3.7. EGF Induced cyclinD1 Upregulation and Cell Cycle Progression through the EGFR/PI3K/Akt Pathway

cyclinD1 is a cell cycle regulatory protein which plays a crucial role in cell proliferation and cell cycle transition from G1 to S phases [26]. To determine the role of cyclinD1 in EGF-induced cell cycle progression, EACKs were incubated with EGF and cyclinD1 protein was detected at different time points by Western blotting analysis. As shown in Figure 5(e), EGF induced cyclinD1 upregulation within 24 h. To investigate the potential contribution of EGFR/PI3K/Akt pathway to cyclinD1 up-regulation, cells were pretreated with AG1478 for 30 min or wortmannin for 1 h and then exposed to EGF. The results demonstrated that both AG1478 and wortmannin significantly inhibited EGF-induced cyclinD1 up-regulation (Figure 5(f)). These findings indicate that EGFR/PI3K/Akt pathway is required for cyclinD1 up-regulation in EGF-stimulated EACKs.

## 4. Discussion

In the current study, we analyzed the protein expression of three key components of the EGFR/PI3K/Akt/cyclinD1 pathway and demonstrated that protein expression of p-EGFR, and p-Akt, cyclinD1 in cholesteatoma epithelium was significantly increased when compared with normal EAC epithelium. In addition, we compared the proliferative capacity of cholesteatoma epithelium with that of normal EAC epithelium, using PCNA as a proliferation marker. We found that cholesteatoma epithelium had a greater proliferative capacity than normal EAC epithelium. Consistent with our observations, previous studies using the Ki-67 proliferation marker also showed a significantly higher proliferation index in cholesteatoma epithelium compared with normal EAC epithelium [1, 27–29]. Moreover, in cholesteatoma epithelium, we demonstrated that a significant positive correlation was observed between p-EGFR and PCNA expression and between the expression levels of p-Akt and PCNA, cyclinD1 and PCNA, respectively. These observations indicate that the EGFR/PI3K/Akt/cyclinD1 pathway is active in cholesteatoma and may be involved in the cellular hyperplasia mechanism in acquired cholesteatoma epithelium.

The EGFR/PI3K/Akt pathway is now recognized as one of the most important pathways in regulating cell survival and proliferation [19, 30]. EGF is one of the most important ligands of the EGFR family. When EGF is present in the extracellular milieu, it can bind to the extracellular domain of EGFR and activate EGFR through autophosphorylation of the intracellular domains of EGFR. Once activated, EGFR results in the activation of PI3K, an intracellular signal transducer enzyme, which can phosphorylate phosphatidylinositol 4,5-biphosphate (PIP2) at the 3′ position of the inositol ring and convert them into phosphatidylinositol 3,4,5-triphosphate (PIP3). As a second messenger, PIP3 recruits Akt to the cell membrane where Akt is fully activated by phosphorylation at position Ser473 [31]. After activation, Akt translocates to the cytoplasm and nucleus to phosphorylate its substrates. For example, Akt has been shown to promote cell proliferative and survival signals through the upregulation of cyclinD1 [32].

To investigate the regulatory mechanisms of EGF as an inducer for the activation of the downstream EGFR/PI3K/Akt/cyclinD1 pathway *in vitro*, we stimulated EACKs with EGF and tested whether EGF induced the cell proliferation of EACKs through the EGFR/PI3K/Akt/cyclinD1 signaling pathway. Our *in vitro* data demonstrated that the stimulation of EACKs with EGF led to the activation of the EGFR/PI3K/Akt signaling pathway, which played a critical role in EGF-induced cell proliferation and cell cycle progression through the up-regulation of cyclinD1. However, both EGFR inhibitor AG1478 and PI3K inhibitor wortmannin significantly inhibited EGF-induced cyclinD1 up-regulation in EACKs. Subsequently, cell proliferation and cell cycle progression of EACKs were also blocked. These results suggest that EGF in the inflammatory microenvironment can phosphorylate and activate EGFR on EACKs. Once activated, EGFR leads to the activation of the downstream PI3K/Akt signaling pathway which subsequently induces cell proliferation and cell cycle progression of EACKs through the up-regulation of cyclinD1. Our observations are consistent with the report by Ouyang et al., in which data was provided that cyclinD1 is a direct down-stream target of the PI3K/Akt signaling pathway [33]. In the study, we found that the EGF effects on the signaling pathway mediators were early; however, the effects on proliferation were seen later 36 h–72 h. As we know, many kinase phosphorylation events occur very quickly after exposure to a stimulus. In our investigation, EGFR and subsequent PI3K/Akt activation have been described as early key events. However, cellular functions such as proliferation which needs mitosis protein synthesis are considered as later final events.

The EGFR/PI3K/Akt signaling pathway is associated with various proliferative lesions including benign and malignant proliferative diseases. Previous studies have revealed that a nonlethal dose of ultraviolet A could promote human HaCat keratinocytes proliferation through the activation of EGFR/PI3K/Akt/cyclinD1 pathway [19]. Recently, it has been reported that a disintegrin and metalloprotease 17 (ADAM17) could induce prostate cancer cell proliferation via EGFR/PI3K/Akt pathway activation [34]. Our results are consistent with these findings and demonstrate for the first time that EGFR/PI3K/Akt/cyclinD1 signaling pathway is active in cholesteatoma epithelium and involved in EGF-induced cell proliferation of EACKs. Therefore, inhibition of the EGFR/PI3K/Akt/cyclinD1 signaling pathway with small molecule inhibitors [19, 35] (e.g., monoclonal antibodies, small molecule tyrosine kinase inhibitors, and small interfering RNA) might be used as a promising target for intratympanic drug therapy for the nonsurgical patients or complementary treatment for preventing recurrence of the cholesteatoma after surgery. In our future study, we will develop animal models of middle ear cholesteatoma in which potential inhibitors of EGFR/PI3K/Akt/cyclinD1 signaling pathway might become more accessible to research.

## 5. Conclusion

In summary, our studies demonstrate that the EGFR/PI3K/Akt/cyclinD1 signaling pathway is active in cholesteatoma and may play a crucial role in cholesteatoma epithelial hyperproliferation. The *in vitro* studies indicate that the EGFR/PI3K/Akt pathway may be involved in EGF-induced cell proliferation of EACKs through the upregulation of cyclinD1. This study may help to understand the molecular mechanisms underlying the pathogenesis of cholesteatoma and identify potential therapeutic targets for intratympanic drug therapy for cholesteatoma.

## Figures and Tables

**Table 1:** The expressions of p-EGFR, p-Akt, cyclinD1, and PCNA in 40 cholesteatoma samples and 20 samples of normal EAC skin.

	p-EGFR			p-Akt			cyclinD1			PCNA		
	+	−	*P *	+	−	*P *	+	−	*P *	+	−	*P *
Cholesteatoma	26	14		29	11		25	15		32	8	
			<0.01			<0.01			<0.01			<0.01
Normal EAC skin	4	16		7	13		2	18		9	11	

**Table 2:** Cell proliferation of EACKs responding to EGF, AG1478, and wortmannin was detected by MTT assay (mean ± SD).

	36 h	48 h	72 h
	Survival rate (%)	Survival rate (%)	Survival rate (%)
Control	100.0	100.0	100.0
EGF	118.2 ± 2.3	132.5 ± 2.8	149.1 ± 3.2
EGF + AG1478	107.8 ± 3.7	108.7 ± 4.3	115.1 ± 2.6
EGF + wortmannin	110.4 ± 2.8	113.1 ± 3.2	118.6 ± 5.2
